# Risk factors for intracerebral hemorrhage in patients with end-stage renal disease on maintenance hemodialysis and establishment of a predictive model

**DOI:** 10.3389/fneur.2026.1770552

**Published:** 2026-07-02

**Authors:** Mengyao Cai, Jiayi Li, Changyun Luo

**Affiliations:** 1 Department of Nephrology, The Affiliated Dazu's Hospital of Chongqing Medical University, Chongqing, China; 2 Department of Nephrology, The People's Hospital of Yubei District of Chongqing, Chongqing, China; 3 Department of Emergency, The Affiliated Dazu's Hospital of Chongqing Medical University, Chongqing, China

**Keywords:** end-stage renal disease, ESRD, intracerebral hemorrhage, maintenance hemodialysis, risk factors

## Abstract

**Background and objective:**

Patients with end-stage renal disease (ESRD) undergoing maintenance hemodialysis (MHD) are at a significantly increased risk of intracerebral hemorrhage (ICH), a devastating complication associated with high mortality and disability. However, reliable tools for early risk assessment remain limited. This study aimed to identify independent risk factors for ICH in this population and to develop a clinically applicable predictive model.

**Methods:**

This is a retrospective, two-center study, 294 ESRD patients undergoing MHD who met the inclusion and exclusion criteria at our hospital and Yubei District People’s Hospital between January 2019 and January 2025 were retrospectively selected as the research objects. Patients were randomly divided into a modeling group (*n* = 206) and a validation group (*n* = 88) in a 7:3 ratio. The modeling group patients were further categorized into a cerebral-hemorrhage group (*n* = 42) and a non-cerebral-hemorrhage group (*n* = 164). Univariate and binary Logistics regression analyses were used to identify the influencing factors for intracerebral hemorrhage in ESRD MHD patients. A predictive model was constructed using SPSS, and R language was used to assess the model’s application value through receiver operating characteristic (ROC) curve, calibration curve, and decision curve analysis (DCA).

**Results:**

The calibration curve slopes in both the modeling group and validation group were close to 1, indicating good consistency between predicted and actual risks. The ROC analysis results revealed an area under the curve (AUC) of 0.96 (95% CI: 0.873–0.979) in the modeling group, while the ROC analysis results revealed an AUC of 0.88(95% CI: 0.793–0.917) in the validation group, demonstrating the model’s effectiveness and clinical benefit in both groups.

**Conclusion:**

This study successfully identified smoking, stroke history, hyperlipidemia, and longer dialysis duration as independent risk factors for ICH in ESRD patients on MHD. The developed prediction model demonstrated good discriminative ability, calibration, and clinical utility in both internal and validation cohorts, providing clinicians with a practical tool for early risk stratification and individualized preventive management. These findings directly address the study’s primary research question and support the model’s potential for clinical implementation pending further external validation.

## 1 Introduction

End-stage renal disease (ESRD), the number of ESRD patients is rapidly increasing at a rate exceeding 10% annually, leading to a continuous expansion of the population undergoing maintenance hemodialysis (MHD) (1). Currently, maintenance hemodialysis is the primary treatment for uremic patients who are not eligible for kidney transplantation. However, while MHD therapy prolongs patient survival and enhances quality of life, it also brings about numerous complications, with intracerebral hemorrhage being particularly severe (2). Cerebrovascular and cardiovascular diseases are significant causes of mortality in chronic dialysis patients, with intracerebral hemorrhage being a leading cause of death among maintenance hemodialysis patients.

Studies have indicated that ESRD is a significant risk factor for intracerebral hemorrhage, which is relatively common in ESRD patients (3). In MHD patients with concomitant stroke, the majority are ischemic, but hemorrhagic strokes still account for about 15%. Risk factors for hemorrhagic stroke in this population include hypertension, bleeding tendencies related to renal failure, and the use of heparin (4). The incidence of intracerebral hemorrhage during maintenance hemodialysis in ESRD patients can be as high as 36.90%. Even with aggressive treatment, the 30-day mortality rate is 30.8%, and the one-year mortality rate is as high as 51.9%. The occurrence of intracerebral hemorrhage not only directly affects the treatment of the primary disease, increasing the complexity of treatment, but also poses a serious threat to patients’ lives, leading to a significant increase in disability and mortality rates. Therefore, the importance of preventing and treating intracerebral hemorrhage in ESRD patients is becoming increasingly apparent (5).

At present, although some studies (6) have explored the risk factors for intracerebral hemorrhage, there is still a lack of systematic and comprehensive analysis as well as effective predictive models (7). Establishing an accurate and reliable predictive model can help clinicians anticipate the risk of intracerebral hemorrhage in advance and adjust treatment plans in a timely manner, thereby improving patient outcomes. Therefore, this study aims to systematically analyze the risk factors for intracerebral hemorrhage in patients with ESRD undergoing MHD and to develop a nomogram predictive model for intracerebral hemorrhage in ESRD MHD patients, providing a scientific basis for the clinical prevention and treatment of intracerebral hemorrhage.

## 2 Research objectives and research methods

### 2.1 Research objectives

**Figure 1:**
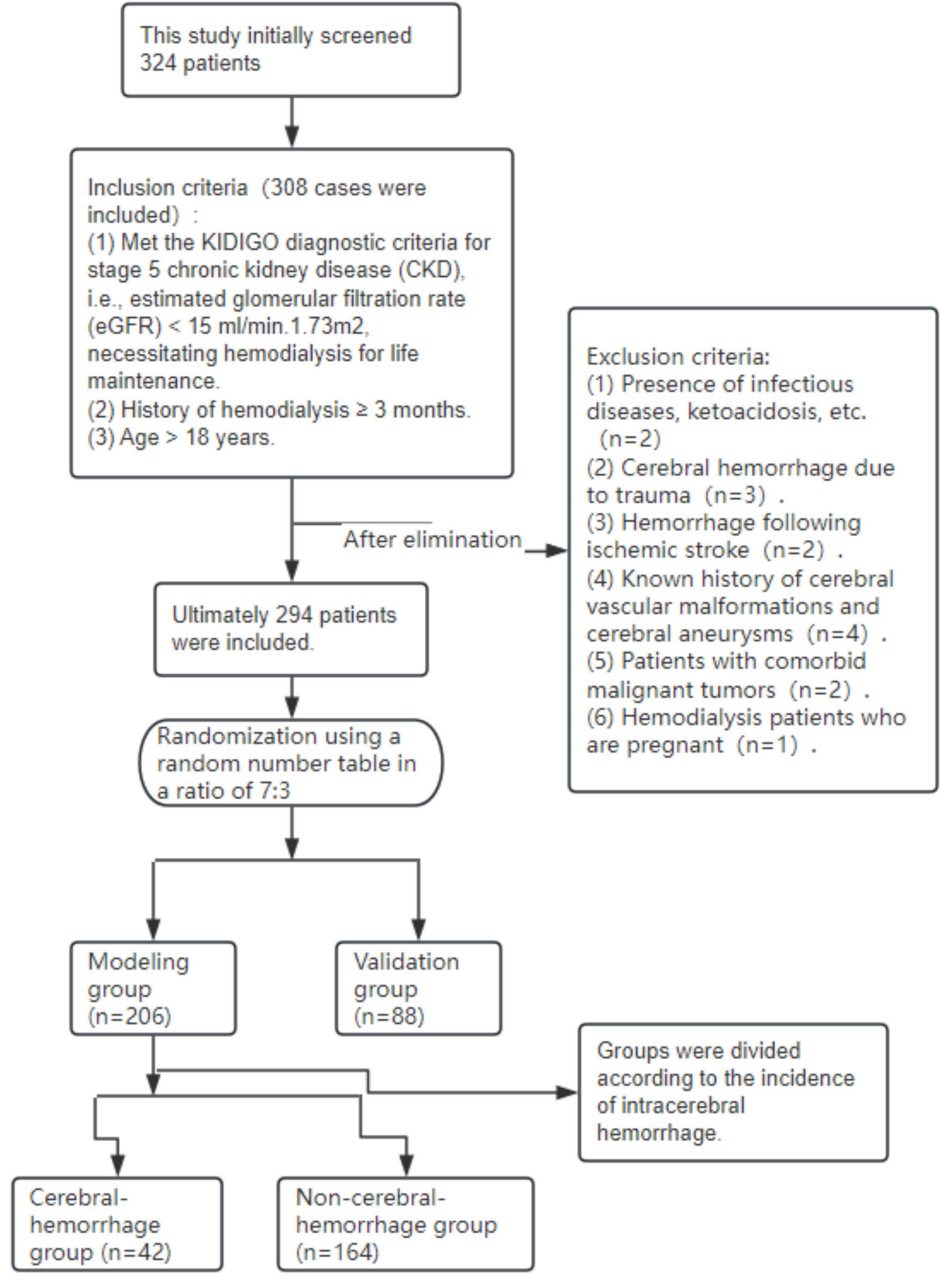
Flowchart.

The sample size was calculated using the formula Z_*α*/2_^2^*π*(1-π)/δ^2^, where π was taken as 15.00% (4) based on previous literature, the significance level α was 0.05, and the allowable error *δ* was 0.06. The calculated sample size was approximately 217. Considering the presence of invalid samples, the sample size was increased by 20% to 260. This study initially screened 324 patients, and ultimately 294 patients were included. The flowchart is shown in Figure 1.

Two hundred ninety four ESRD patients undergoing MHD at our hospital and Yubei District People’s Hospital, who met the inclusion and exclusion criteria between January 2019 and January 2025, were retrospectively selected as study subjects. Inclusion criteria: (1) Met the KIDIGO diagnostic criteria for stage 5 chronic kidney disease (CKD), i.e., estimated glomerular filtration rate (eGFR) < 15 mL/min.1.73m^2^, necessitating hemodialysis for life maintenance (6). (2) History of hemodialysis ≥ 3 months. (3) Age > 18 years. Exclusion criteria: (1) Presence of infectious diseases, ketoacidosis, etc. (2) intracerebral hemorrhage due to trauma. (3) Hemorrhage following ischemic stroke. (4) Known history of cerebral vascular malformations and cerebral aneurysms. (5) Patients with comorbid malignant tumors. (6) Hemodialysis patients who are pregnant. This study followed TRIPOD and STROBE guidelines.

### 2.2 Research methods

#### 2.2.1 Grouping

Patients were randomly divided in a 7:3 ratio using a random number table method into a modeling group (*n* = 206) and a validation group (*n* = 88). Within the modeling group, patients were further categorized based on the occurrence of intracerebral hemorrhage into a cerebral-hemorrhage group (*n* = 42) and a non-cerebral-hemorrhage group (*n* = 164). Diagnostic criteria: All patients with intracerebral hemorrhage had imaging evidence, such as cranial CT and/or magnetic resonance imaging (7).

#### 2.2.2 Data collection

By reviewing literature and data and combining professional knowledge, potential predictive factors were selected and collected. These factors include age, gender, BMI, education level, smoking, alcohol consumption, medical history (hypertension, diabetes, hyperlipidemia, history of stroke, duration of dialysis, dialysis modality, history of oral anticoagulant use, antiplatelet drug use, history of nonsteroidal anti-inflammatory drug use, etc.), laboratory parameters (complete blood count, albumin, parathyroid hormone, C-reactive protein, coagulation analysis, calcium, phosphorus, etc.), and more.

In this study, missing values were handled using deletion or imputation methods. If the proportion of missing data is very small, the missing values are either retained in subsequent analysis and modeling, or filled using statistical measures such as the mean or median. This helps maintain the integrity of the dataset. If a feature has a large amount of missing data and is deemed of low importance for the analysis objective based on previous references, the feature is deleted.

### 2.3 Statistical analysis

The experimental data collected were analyzed using SPSS 27.0 (International Business Machines Corporation, Armonk, New York, USA). The Shapiro–Wilk test was employed for normality testing. For normally distributed continuous data in the experimental data, results were expressed as^−^X ± S. Independent sample *t*-tests were used for comparisons, while multiple group comparisons were conducted using *F*-tests. Categorical data were presented as counts or rates, and comparisons were performed using *χ*^2^ test or Fisher’s exact test. Bring meaningful variables from the single factor analysis into the multivariate analysis,univariate and binary Logistics regression analyses were conducted to determine the influencing factors of intracerebral hemorrhage in ESRD MHD patients. A predictive model was constructed using SPSS, and R language4.0 was utilized to generate receiver operating characteristic (ROC) curves, calibration curves, and decision curve analysis (DCA) to assess the model’s application value. A significance level of *p* < 0.05 was considered statistically significant for differences.

Rationale for the statistical approach: First, univariate analysis screened potential risk factors for intracerebral hemorrhage (variables with *p* < 0.10 entered multivariate binary logistic regression). This stepwise approach reduces confounder omission and overfitting. Binary logistic regression was used as the outcome is binary, enabling adjustment of multiple covariates to identify independent risk factors. A predictive model was built based on these factors, using regression coefficients for a linear prediction equation. A nomogram was developed for clinical use, converting complex equations into an intuitive scoring system. ROC curves evaluated model discrimination (AUC closer to 1 is better), calibration curves assessed predicted-observed agreement (slope close to 1 indicates good calibration), and DCA evaluated clinical net benefit across thresholds, following TRIPOD guidelines. SPSS (27.0) was used for regression modeling, and R (4.0) for graphical validation and DCA.

## 3 Results

### 3.1 Univariate analysis of factors influencing intracerebral hemorrhage in ESRD MHD patients

Comparison of general information between the modeling group and validation group patients showed no statistically significant differences. In the modeling group, comparisons between the cerebral-hemorrhage group and the non-cerebral-hemorrhage group revealed statistically significant differences (*p* < 0.05) in such factors as smoking, history of stroke, hyperlipidemia, duration of dialysis, use of antiplatelet drugs, BNP levels, fibrinogen levels, and blood sodium levels; see Table 1 for details.

**Table 1:** Univariate analysis of factors influencing intracerebral hemorrhage in ESRD MHD Patients.

Baseline data	Category	Modeling Group (*n* = 206)		Validation group (*n* = 88)
		Cerebral-hemorrhage group (*n* = 42)	Non-cerebral-hemorrhage group (*n* = 164)	
Age (years)		52.88 ± 11.05	53.12 ± 10.41	52.87 ± 10.82
Gender	Male	22	86	48
	Female	20	78	40
BMI (kg/m^2^)		21.35 ± 1.25	21.44 ± 1.13	21.47 ± 1.26
Education level	Below College	24	92	51
	College and above	18	72	37
Smoking	Yes	19*	43	21
	No	23	121	67
Alcohol drinking	Yes	16	65	39
	No	26	99	49
Hypertension	Yes	30	114	63
	No	12	50	25
Diabetes	Yes	25	98	57
	No	17	66	31
History of stroke	Yes	22*	34	26
	No	20	130	62
Hyperlipidemia	Yes	20*	42	30
	No	22	122	58
Duration of dialysis (years)		7.54 ± 1.12*	5.68 ± 1.21	6.43 ± 1.37
Dialysis modality	Hemodialysis	34	124	70
	Hemodialysis	8	40	18
Use of oral anticoagulant	Yes	9	21	17
	No	33	143	71
Use of antiplatelet drug	Yes	26*	61	42
	No	16	103	46
Use of NSAID	Yes	15	55	33
	No	27	109	55
WBC (10^9^/L)		8.17 ± 2.21	7.93 ± 2.54	8.62 ± 3.34
PLT (10^9^/L)		166.64 ± 43.54	176.82 ± 43.21	163.82 ± 41.67
SCR (μmol/L)		521.62 ± 100.87	493.33 ± 166.22	513.25 ± 121.82
BUN (mg/dL)		16.87 ± 5.14	17.21 ± 5.26	16.99 ± 5.24
TG (mmol/L)		2.24 ± 0.78	2.34 ± 0.89	2.26 ± 0.92
TC (mmol/L)		4.43 ± 0.87	4.56 ± 0.92	4.53 ± 0.87
LDL-c (mmol/L)		3.12 ± 0.92	3.08 ± 0.97	3.11 ± 0.87
HDL (mmol/L)		1.35 ± 0.34	1.41 ± 0.44	1.32 ± 0.37
BNP (pg/ml)		628.65 ± 127.32*	483.54 ± 103.54	537.81 ± 119.76
CRP (mg/L)		7.33 ± 2.24	8.01 ± 3.12	7.62 ± 3.11
Prothrombin time (s)		10.67 ± 4.32	11.22 ± 3.97	11.06 ± 3.17
D-dimer (ug/L)		1.46 ± 0.38	1.37 ± 0.29	1.41 ± 0.43
Fibrinogen (g/L)		5.20 ± 1.42*	3.27 ± 1.36	4.19 ± 1.28
Blood calcium (mmol/L)		2.42 ± 0.49	2.39 ± 0.31	2.44 ± 0.28
Blood phosphorus (mmol/L)		1.88 ± 0.29	1.84 ± 0.31	1.88 ± 0.46
Blood sodium (mmol/L)		131.82 ± 3.26*	140.54 ± 3.89	136.84 ± 3.43

“*” indicates a statistically significant difference compared to the non-cerebral-hemorrhage group.

### 3.2 Binary logistics regression analysis of factors influencing intracerebral hemorrhage in ESRD MHD patients

Using the significant variables identified in the univariate analysis as independent variables, as shown in Table 2, a binary Logistics regression analysis was conducted with intracerebral hemorrhage as the dependent variable (occurred = 1, not occurred = 0). The results of the binary Logistics regression analysis indicated that smoking (OR = 2.254, 95%CI:1.005 ~ 5.054), history of stroke(OR = 3.156, 95%CI:1.194 ~ 8.343), hyperlipidemia(OR = 3.228, 95%CI:1.280 ~ 8.142), and duration of dialysis(OR = 2.542, 95%CI:1.379 ~ 4.685) were significant influencing factors for intracerebral hemorrhage in ESRD MHD patients (*p* < 0.05), as shown in Table 3.

**Table 3:** Binary logistics regression analysis results.

Variable	*B*	SE	Wald	*p*	Exp(B)	95%CI
Smoking	0.813	0.412	3.891	0.041	2.254	1.005 ~ 5.054
History of stroke	1.149	0.496	5.369	0.001	3.156	1.194 ~ 8.343
Hyperlipidemia	1.172	0.472	6.164	<0.001	3.228	1.280 ~ 8.142
Duration of dialysis	0.933	0.312	8.941	<0.001	2.542	1.379 ~ 4.685
Use of antiplatelet drug	1.002	0.526	3.632	0.302	2.725	0.972 ~ 7.640
BNP	0.684	0.377	3.293	0.414	1.982	0.947 ~ 4.150
Fibrinogen	0.630	0.369	2.917	0.712	1.878	0.911 ~ 3.871
Blood sodium	0.422	0.338	1.559	0.078	1.525	0.786 ~ 2.958

### 3.3 Establishment of a predictive model

**Figure 2:**
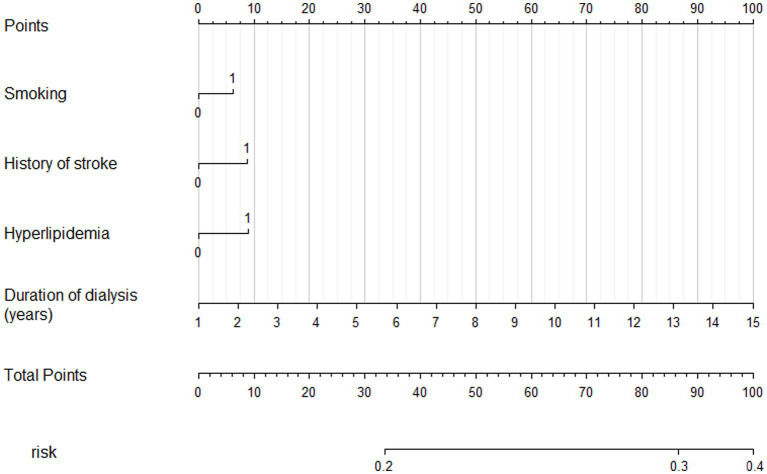
Nomogram model.

**Figure 3:**
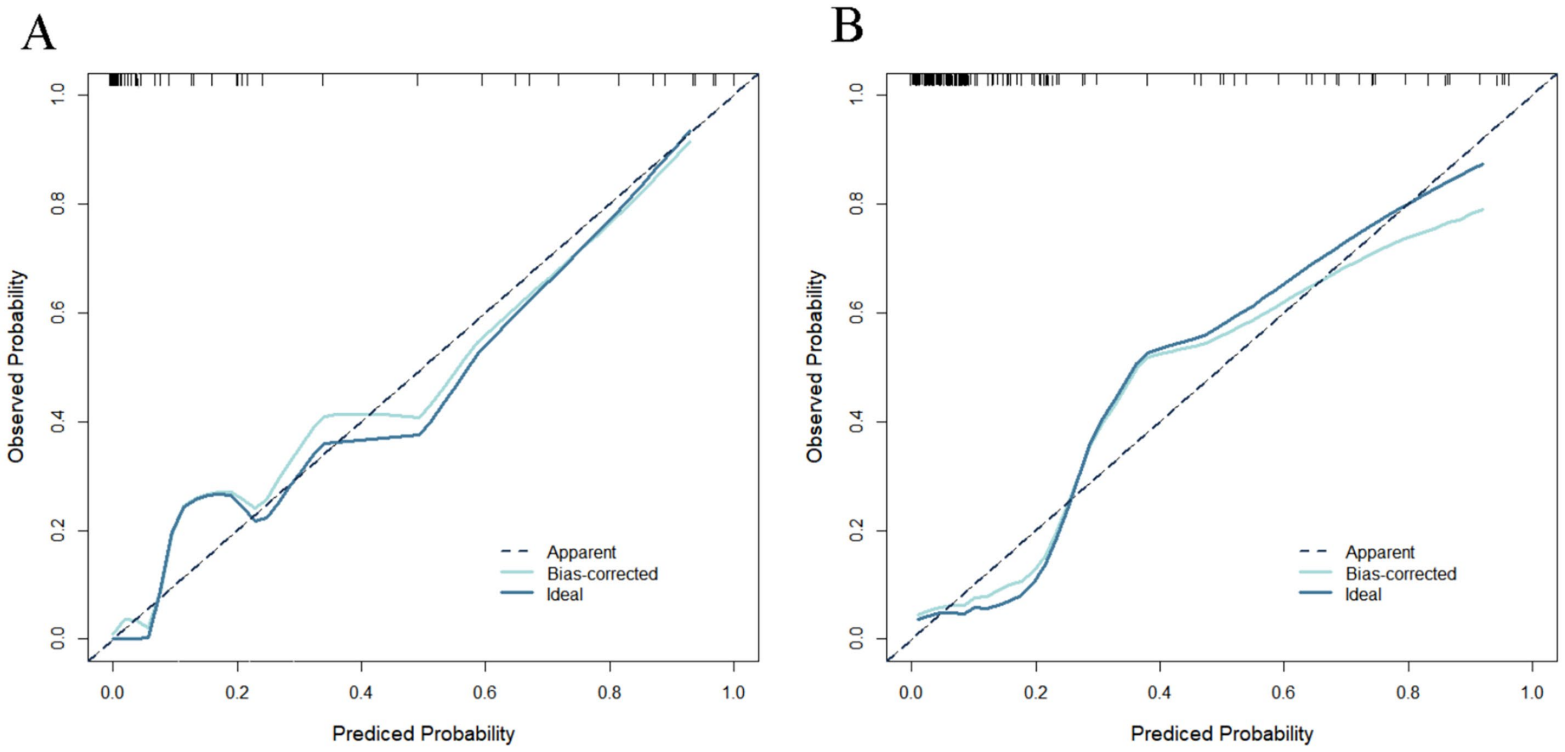
Calibration curves. **(A)** shows the calibration curve for the modeling group, while **(B)** displays the calibration curve for the validation group. The curves are close to a slope of 1, indicating good consistency between the predicted and actual risks.

Based on the results of the Logistics regression analysis, smoking, history of stroke, hyperlipidemia, and duration of dialysis were included in the constructed predictive model. The expression of the combined detection factor model is Logit(P) = 2.182 + (0.813*Smoking) + (1.149*History of Stroke) + (1.172*Hyperlipidemia) + (0.933*Duration of Dialysis). The nomogram is as shown in Figure 2. The calibration curves of this model in both the modeling group and validation group display a slope close to 1, indicating good consistency between the predicted and actual risks. See Figure 3 for details.

### 3.4 ROC curve analysis

**Figure 4:**
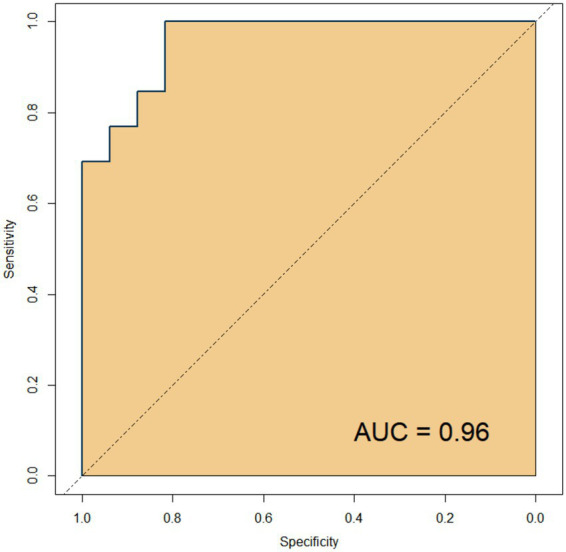
ROC curve of modeling group.

**Figure 5:**
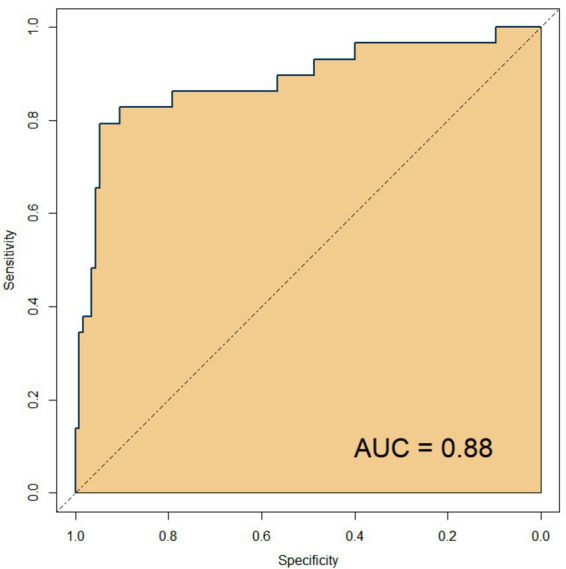
ROC curve of validation group.

The ROC analysis results indicated that the model’s area under the curve (AUC) in the modeling group was 0.96 with a standard error (SE) of 0.021 (95% CI: 0.873–0.979) and a Youden index of 0.81. At this point, the sensitivity was 80.54%, and the specificity was 100.00%, as shown in Figure 4. In the validation group, the model’s AUC was 0.88 with a SE of (95% CI: 0.793–0.917) and a Youden index of 0.72. At this threshold, the sensitivity was 91.87%, and the specificity was 79.82%, as depicted in Figure 5.

### 3.5 Clinical benefit analysis of predictive model

**Figure 6:**
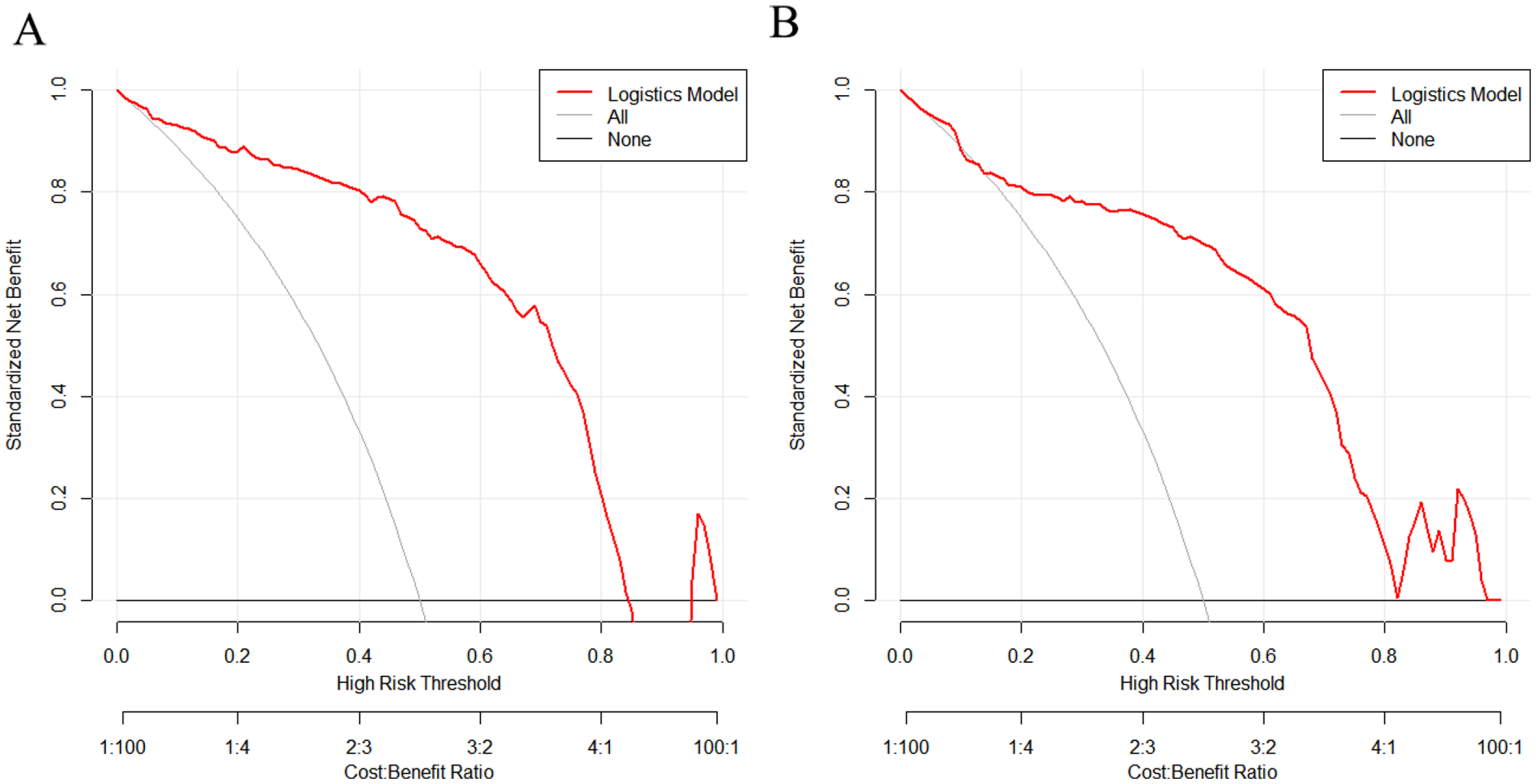
DCA curve. **(A)** shows the DCA curve for the modeling group, while **(B)** displays the DCA curve for the validation group.

To assess the clinical utility of the model in predicting therapeutic effects, DCA curves were plotted. The “net benefit rate” was used to balance the pros and cons of accurately treating high-risk patients and avoiding overtreatment of low-risk patients. In the DCA curve, the Y-axis represents the net benefit rate for patients based on the model’s decision. The red curve is drawn based on the model’s predicted target population compliance, while the black and gray horizontal lines represent the extreme scenarios of no patients and all patients experiencing intracerebral hemorrhage, respectively. A lower value is indicated when the red curve is closer to the black and gray lines, and better clinical benefits are achieved when it is closer to the top right corner. This study demonstrates that the model shows good application effectiveness and clinical benefits in both groups, as illustrated in Figure 6.

## 4 Discussion

This study focused on ESRD MHD patients, investigating the risk factors for intracerebral hemorrhage and constructing a predictive model. Patients with ESRD often rely on maintenance hemodialysis to sustain life due to renal function failure and metabolic disorders. However, during hemodialysis, patients are at risk of various complications, with intracerebral hemorrhage being a severe complication that can lead to patient mortality. Understanding the risk factors for intracerebral hemorrhage can help clinicians identify high-risk patients early, implement targeted preventive measures, and consequently reduce the incidence of intracerebral hemorrhage, thereby improving patient quality of life and prognosis. Through univariate and binary Logistics regression analyses, this study found that smoking, history of stroke, hyperlipidemia, and duration of dialysis are influencing factors for intracerebral hemorrhage in ESRD patients undergoing maintenance hemodialysis. Based on the risk factors mentioned above, the predictive model constructed in this study provides clinicians with an effective tool to predict the risk of intracerebral hemorrhage in ESRD patients undergoing maintenance hemodialysis.

Smoking, as a harmful lifestyle habit, exposes the endothelium to damaging substances like nicotine and tar, leading to vascular endothelial damage, decreased vascular elasticity, and thickening of vessel walls, increasing the risk of cerebral vascular rupture (5). For ESRD patients, whose vascular function is already compromised, smoking undoubtedly exacerbates this damage, thereby increasing the likelihood of intracerebral hemorrhage occurrence. A history of stroke is also a significant risk factor for intracerebral hemorrhage. Patients with a history of stroke often have pre-existing cerebrovascular lesions such as atherosclerosis and vascular stenosis (8). These lesions render the cerebral vasculature more fragile, making them more susceptible to rupture during hemodialysis due to factors like blood pressure fluctuations and changes in coagulation function (9, 10). Therefore, ESRD patients undergoing maintenance hemodialysis with a history of stroke require closer monitoring of their cerebral vascular status and timely adjustment of treatment plans. Hyperlipidemia leads to lipid metabolism disorders in the blood, with excessive lipids depositing on vessel walls to form atherosclerotic plaques. These plaques narrow the vessel lumen, impeding blood flow and reducing vessel elasticity (11). During hemodialysis, changes in blood flow velocity and pressure may cause unstable plaques to rupture, leading to thrombus formation or vascular rupture (12). Thus, controlling hyperlipidemia is crucial for preventing intracerebral hemorrhage in ESRD patients undergoing maintenance hemodialysis. The duration of dialysis is closely related to the occurrence of intracerebral hemorrhage. With prolonged dialysis time, patients are in a prolonged non-physiological state, exposing their vessels to repeated stimulation and injury (13). Additionally, medications such as anticoagulants used during dialysis may impact coagulation function, increasing the risk of bleeding (14). Furthermore, long-term dialysis patients often have multiple comorbidities like anemia and malnutrition, further weakening their body’s resistance and making them more susceptible to intracerebral hemorrhage.

**Table 2:** Variable assignment.

Influencing factor	Assignment
Smoking	No = 0, Yes = 1
History of stroke	No = 0, Yes = 1
Hyperlipidemia	No = 0, Yes = 1
Duration of dialysis	Original value
Use of antiplatelet drug	No = 0, Yes = 1
BNP	Original value
Fibrinogen	Original value
Blood sodium	Original value

Previous studies by Zheng et al. (15) have shown a significant association between smoking, hyperlipidemia, and Cerebral Small Vessel Disease(CSVD) in ESRD patients, where CSVD is an important risk factor for intracerebral hemorrhage, thus indirectly supporting the results of this study. Previous models (16) on heart failure in MHD patients or were limited to the predictive value of single indicators (17), lacking researches on predictive models for intracerebral hemorrhage in ESRD MHD patients. The results of this study further provide clinical references in this specific area. The results of this study complement Li et al. (5), multicenter machine learning study, which revealed the predictive value of blood lipid indicators such as LDL and HDL for cerebral hemorrhage using an XGBoost model. This is highly consistent with the ‘hyperlipidemia’ risk factors identified through traditional regression analysis in our study. Moreover, the mechanistic pathways elaborated in our study provide a pathophysiological explanation for inflammatory markers such as CRP, which were not parsed in Li′s model. It is worth noting that the outstanding performance of Li′s model, with an AUC of 0.979, may be attributed to the inclusion of temporal data such as dynamic blood pressure monitoring. In contrast, our study used baseline blood pressure (SBP OR = 1.933, 95%CI 1.317–2.836), suggesting that future hybrid models integrating continuous blood pressure data from wearable devices may overcome the limitations of static prediction.

By inputting relevant patient information such as smoking status, history of stroke, presence of hyperlipidemia, and duration of dialysis, the model can calculate the probability of a patient experiencing intracerebral hemorrhage. This assists clinicians in early intervention for high-risk patients, such as adjusting dialysis regimens, controlling blood pressure and lipid levels, and enhancing anticoagulant therapy management, thereby reducing the incidence of intracerebral hemorrhage. From the evaluation metrics of the model, it exhibited good performance in both the modeling and validation groups. The calibration curves with slopes close to 1 indicate good consistency between predicted and actual risks, meaning the model can accurately predict the probability of patients experiencing intracerebral hemorrhage. The ROC analysis results indicated that the model had a relatively large area under the curve in both the modeling and validation groups, demonstrating good discriminative ability to effectively distinguish patients who experienced intracerebral hemorrhage from those who did not. Additionally, the sensitivity and specificity were at high levels, indicating good accuracy in identifying high-risk patients and excluding low-risk patients. Further validation through DCA confirmed the clinical utility of the model. Clinicians can use the model’s predictive results to develop appropriate treatment strategies, avoiding unnecessary overtreatment of low-risk patients while ensuring timely and effective interventions for high-risk patients.

While this study has achieved certain results, there are still some limitations. Firstly, this study is a single-center retrospective study with a relatively limited sample size, which may introduce selection bias. ESRD patients undergoing maintenance hemodialysis from different regions or hospitals may have variations in underlying diseases and treatment plans, making it challenging to generalize the results of this study to other populations. Although this study initially confirmed the robustness of the prediction model through strong verification (AUC0.88), there are still important limitations that need to be noted: during the variable screening stage, multiple test adjustments (such as Bonferroni or FDR corrections) were not performed on about 30 candidate variables, resulting in a significant increase in the risk of type I error inflation. Although this issue was partially alleviated by strict inclusion criteria (such as retaining only *p* < 0.05 variables) and independent validation sets (*n* = 88), potential false positive associations in high-dimensional data may still affect model extrapolation. Future research needs to combine machine learning variable selection methods or cross-validation strategies to more strictly control the false discovery rate and improve the credibility of prediction efficiency.

Therefore, future researches should focus on multi-center, prospective studies to increase the sample size and enhance the universality and reliability of the research findings. Secondly, although this study considered some common clinical indicators in analyzing risk factors, there may be other unidentified risk factors that could impact the occurrence of intracerebral hemorrhage in ESRD patients undergoing maintenance hemodialysis. Future studies could further explore these potential risk factors to refine the variable selection of predictive models. Additionally, the predictive model constructed in this study has only been validated in the study population, and its effectiveness in actual clinical practices requires further observation and evaluation. Clinicians using this model should make comprehensive judgments based on the specific conditions of individual patients and not solely rely on the predictive results of the model. Furthermore, as medical technology advances and understanding of diseases deepens, predictive models need continuous updates and enhancements to improve their predictive accuracy and clinical utility.

## 5 Conclusion

In conclusion, this study analyzed the risk factors for intracerebral hemorrhage in ESRD MHD patients and established a predictive model, providing a basis for clinical prevention and treatment of intracerebral hemorrhage. However, the study still has limitations, and further high-quality researches are needed to validate and improve the model. The risk prediction model constructed in this study can quantify the risk of cerebral hemorrhage in patients with end-stage renal disease on hemodialysis by integrating key variables such as smoking and stroke history, and provides an early decision-making tool for clinical formulation of individualized prevention strategies.

## Data Availability

The original contributions presented in the study are included in the article/supplementary material, further inquiries can be directed to the corresponding author.
